# Analyses of Kaposi Sarcoma trends among adults establishing initial outpatient HIV care in Nigeria: 2006–2017

**DOI:** 10.1186/s13027-022-00424-4

**Published:** 2022-03-21

**Authors:** Maxwell O. Akanbi, Lucy A. Bilaver, Chad Achenbach, Lisa R. Hirschhorn, Adovich S. Rivera, Olugbenga A. Silas, Patricia A. Agaba, Oche Agbaji, Nathan Y. Shehu, Solomon A. Sagay, Lifang Hou, Robert L. Murphy

**Affiliations:** 1 Department of Internal Medicine, McLaren Hospital, 401 S Ballenger Hwy, Flint, MI 48532 USA; 2 Health Sciences Integrated Ph.D. Program, Center for Education in Health Sciences, Institute for Public Health and Medicine, Feinberg School of Medicine, Northwestern University, Chicago, USA; 3 Robert J Havey Institute for Global Health, Center for Global Communicable Diseases, Feinberg School of Medicine, Northwestern University, Chicago, USA; 4 Department of Medicine, College of Health Sciences, University of Jos, Jos, Nigeria; 5 Department of Infectious Diseases, Northwestern University Feinberg School of Medicine, Chicago, USA; 6 Department of Medical Social Sciences, Northwestern University Feinberg School of Medicine, Chicago, USA; 7 Department of Pathology, College of Human Sciences, University of Jos, Jos, Nigeria; 8 Department of Family Medicine, College of Human Sciences, University of Jos, Jos, Nigeria; 9 Department of Obstetrics and Gynecology, College of Human Sciences, University of Jos, Jos, Nigeria; 10 Department of Prevention Diseases (Cancer Epidemiology and Prevention), Northwestern University Feinberg School of Medicine, Chicago, USA; 11 Institute for Global Health, Center for Global Oncology, Feinberg School of Medicine, Northwestern University, Chicago, USA

**Keywords:** Africa, Antiretroviral therapy, Epidemiology, Human Immunodeficiency Virus, Kaposi Sarcoma

## Abstract

**Background:**

The incidence of Human Immunodeficiency Virus (HIV)-associated Kaposi Sarcoma (KS) in the pre-antiretroviral therapy (ART) population remains high in several countries in sub-Saharan Africa. We examined trends of KS prevalence in adults, establishing initial outpatient HIV care from 2006 to 2017 in Nigeria.

**Methods:**

We analyzed data of 16,431 adults (age ≥ 18 years) enrolled for HIV care from January 1, 2006, to December 31, 2017, in a large clinic in Jos, Nigeria. KS at enrollment was defined as KS recorded in the electronic health record within 30 days of clinic enrollment. Time trends were compared among four periods: 2006–2008, 2009–2011, 2012–2014, and 2015–2017 using logistic regression models. Annual trends were analyzed using join point regression and restricted splines.

**Results:**

The study population had a mean age 35.1 (standard deviation, SD 9.5) years, and were 65.7% female (n = 10,788). The mean CD4 cell count was 220 (95% CI 117–223). The overall KS prevalence at entry was 0.59% (95% CI 0.48–0.72). Compared to 2006–2008, KS prevalence was significantly higher in 2009–2011 (adjusted odds ratio 5.07 (95% CI 3.12–8.24), *p* < 0.001), but remained unchanged in subsequent periods. Male sex and low CD4 T-cell count independently increased odds for KS.

**Conclusions:**

Despite ART expansion, KS at enrollment showed no significant decline. The low CD4 cell count, across all periods, indicates delay in enrollment for HIV care, which increases KS risk. Interventions aimed at early HIV diagnosis and linkage to ART is critical to KS risk reduction in this population.

**Supplementary Information:**

The online version contains supplementary material available at 10.1186/s13027-022-00424-4.

## Introduction

Kaposi Sarcoma (KS) is the malignancy that most strongly mirrors the Human Immunodeficiency Virus (HIV) epidemic [1–3]. In most Western countries, following an initial surge in KS cases at the outset of the HIV epidemic, the emergence of combination antiretroviral therapy (ART) in the late 1990s led to a dramatic decline in KS cases among people living with HIV (PLWH) [1, 4]. However, in sub-Saharan Africa (SSA), after nearly 2 decades of ART, KS remains a major cause of morbidity and mortality [5, 6]. Of 42,000 incident cases and 20,000 deaths attributable to KS worldwide in 2018, SSA was responsible for 60% of the incident cases and 90% of the deaths [5]. PLWH with KS have a higher mortality risk, loss-to-follow-up, and poorer immunologic response compared to those without KS [7, 8].

In the last decade, countries in SSA have witnessed a significant expansion of their HIV programs [9, 10]. The most significant gain has been improving ART coverage, which has exceeded 80% in several countries [11]. Still, prompt HIV diagnosis and linkage to care remains a weak link in the HIV care cascade in SSA [12–14]. While timely HIV diagnosis and prompt initiation of ART has a great impact on the occurrence of HIV-associated KS, the impact of HIV program expansion on KS risk among patients initiating HIV care in SSA remains unclear.

With 1.7 million people living with HIV by the end of 2020, Nigeria has the second largest HIV population in the world [15, 16]. KS was relatively uncommon in Nigeria before the HIV pandemic, but is now being reported as the most common malignancy in males in certain sub-populations of Nigeria [17]. Nigeria is one of the countries in SSA that has significantly expanded its HIV program, with the most significant gains being the expansion of ART coverage. From 2006 to 2020, ART coverage increased from 10 to 86%, respectively [11]. However, less progress has been made in prompt HIV diagnosis and linkage to ART. The 2018 Nigeria HIV-AIDS Indicator and Impact Survey (NAIIS) revealed that while 96% of Nigerians who were aware that they had HIV were receiving ART, with 86% of this population virally suppressed, only 47% of people living with HIV were aware of their status [18]. With this in mind, the impact of Nigeria’s HIV program expansion on KS risk in the pre-ART population is unclear.

Utilizing longitudinal data from one of Nigeria’s largest HIV clinic cohorts, we describe trends in annual KS prevalence among adults establishing initial outpatient HIV care from 2006 to 2017.

## Methods

### Study design and setting

We analyzed data of PLWH who enrolled for initial HIV outpatient care at one of the largest stand-alone HIV treatment facilities in Nigeria. The clinic commenced patient enrollment in 2002, and by December 2017, had provided care to over 30,000 persons with HIV. HIV care and treatment were supported by funding from the Government of Nigeria, the Bill and Melinda Gates Foundation, and the United States President’s Emergency Plan for AIDS Relief (PEPFAR) fund [19, 20]. Follow-up visits were scheduled monthly or every other month. During patient visits, data on AIDS-defining events, including KS, was routinely obtained.

### Conceptual framework

**Fig. 1:**
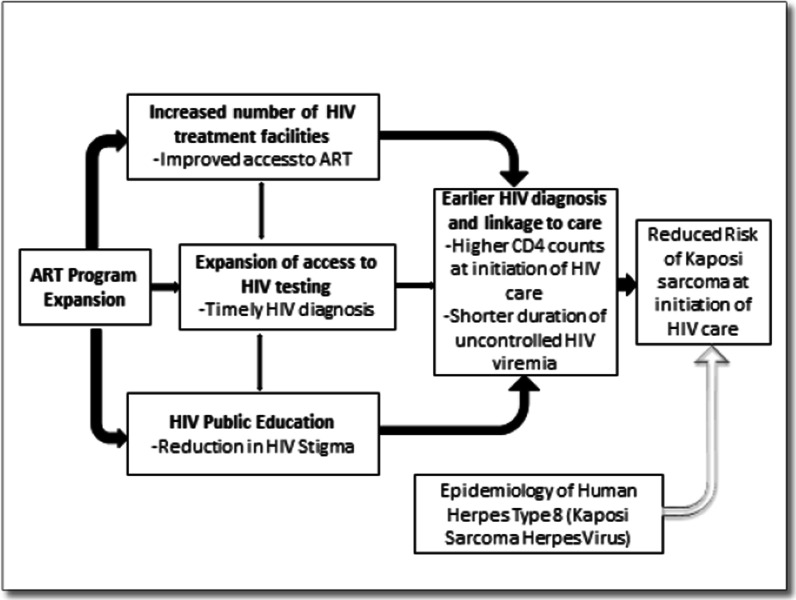
Conceptual model of the impact of HIV program expansion on Kaposi Sarcoma risk in patients initiating HIV care

The conceptual framework for our study is shown in Fig. 1. We posit that the expansion of HIV treatment programs could influence KS risk among patients newly engaging in HIV care through interventions aimed at facilitating prompt HIV diagnosis and linkage to care (the early parts of the HIV care continuum) [13]. These interventions include public education to reduce HIV stigma and facilitate self-referral for HIV testing. Key interventions also include the expansion of access to HIV testing, such as community-based HIV testing programs and HIV-self testing, and the linkage of newly diagnosed HIV patients to ART [21]. Prompt HIV diagnosis and linkage to ART prevents the development of profound HIV-induced immunosuppression, which is the strongest modifiable risk factor for KS [22, 23]. Although infection with the Human Herpes Virus Type 8 (HHV-8) is necessary for the development of KS, there is limited information on its mode of transmission, and there is currently no vaccine or treatment for it [24, 25].

### Data sources and study variables

We utilized de-identified, routinely collected data stored in the clinic’s electronic medical record (EMR). The EMR was developed and maintained through a program grant awarded to the Harvard School of Public Health [20].

The study included PWH 18 years and older, all who were ART-naive and enrolled for their initial HIV out-patient care from January 1, 2006, through December 31, 2017. We excluded records of PWH if they did not complete the clinic enrollment process, and thus have missing HIV confirmation and/or KS outcome data (n = 2686), or were younger than 18 years at clinic enrollment (n = 81).

As per the clinic protocol, at the first clinic encounter, data was systematically collected using a standardized “Pre-assessment Form” and transferred into the EMR. Routinely collected data included: age (years), sex (male or female), HIV risk factors (heterosexual, intravenous drug use, transfusion, men who had sex with men, mother to child HIV transmission, and unknown), previous ART use (Yes/No), and CD4 T-cell count. The presence of HIV-related diagnoses, including KS, was reported as “yes” if present and “no” if absent.

In our analyses, we defined KS at enrollment as KS diagnosed within 30 days of clinic enrollment [4]. Two clinic visits within 30 days were required to complete the enrollment process. The first visit was dedicated to HIV counseling, health education, and HIV confirmation. At the second visit, patients who were confirmed to have HIV had blood samples taken for CD4 count, as well as evaluation for opportunistic diseases (including KS) to determine eligibility for ART, based on the current National HIV treatment guidelines. Patients who did not show up for the second visit were classified as having “incomplete data”. The quality of our data was enhanced by ongoing NIH-sponsored HIV and cancer research, focused on validating cancer cases in the EMR.

### Statistical methods

Characteristics of the study population were summarized using descriptive statistics. To increase the power of the study, the 12-year study period was divided into four periods: 2006–2008, 2009–2011, 2012–2014, and 2015–2017. To minimize bias due to missing variables, we used the multiple imputation method (20 imputations), based on Rubin’s method, to impute missing baseline CD4 T-cell count, which was the only covariate with missing values (n = 1645) [26]. The crude prevalence of KS was computed per 1000 population. We used logistic regression to examine changes in KS prevalence over the four periods, with post-hoc all-way pairwise comparison. Margin plots were used to display changes in KS prevalence over time by age group (< 30, 30–39, and ≥ 40 years) and sex.

For sensitivity analyses, we evaluated annual changes in KS prevalence using restricted cubic splines analysis and join point regression. Because our conceptual model predicts that changes in KS are dependent on CD4 T-cell count at enrollment, we also modeled annual changes in CD4 T-cell count at enrollment over time using restricted cubic splines. Data analyses were carried out using Stata Statistical Software: Release 14. College Station, TX: Stata Corp LP, SAS, and R 4.0/RStudio. In all analyses, a two-sided *p* < 0.05 was considered significant.

The study was approved by the local Institutional Review Board (IRB), and it was ruled exempt by the IRBs of Northwestern University and Harvard School of Public Health.

## Results

### Demographic characteristics

We analyzed the data of 16,431 patients, with a mean age of 35 (standard deviation (SD) 10) years and 65.7% female (n = 10,788). The mean CD4 T-cell count at enrollment was 220 (95% CI 117–223) cells/mm^3^. CD4 T-cell count at enrollment was lowest for patients who initiated HIV care in 2006–2008 (210; 95% CI 206–213 cells/mm^3^) (Table 1).

**Table 1:** Characteristics of adults who initiated HIV care between 2006 and 2017 in Jos, Nigeria

Patient characteristics	Period of enrollment				
	Total (N = 16,431)	2006–2008 (N = 9357)	2009–2011 (N = 3329)	2012–2014 (N = 2449)	2015–2017 (N = 1296)
Age, years, means (SD)	35.1 (9.5)	34.9 (9.3)	34.8 (9.2)	35.7 (9.9)	35.9 (10.6)
Age group, years, n (%)					
< 30	5025 (30.6)	2888 (30.86)	1047 (31.5)	723 (29.5)	367 (28.3)
30–39	6598 (40.2)	3811 (40.73)	1370 (41.2)	931 (38.0)	486 (37.5)
≥ 40	4808 (29.3)	2658 (28.4)	912 (27.4)	795 (32.5)	443 (34.2)
Sex, n (%)					
Female	10,788 (65.7)	6263 (66.9)	2173 (65.3)	1,564 (63.9)	788 (60.8)
Male	5643 (34.3)	3094 (33.1)	1156 (34.7)	885 (36.1)	508 (39.2)
Risk for HIV					
Heterosexual	16,155 (98.3)	9203 (98.4)	3240 (97.3)	2435 (99.4)	1277 (98.5)
Blood transfusion	184 (1.1)	139 (1.5)	25 (0.8)	14 (0.6)	6 (0.5)
Others^a^	92 (0.56)	15 (0.2)	64 (1.9)	0 (0.0)	13 (1.0)
Baseline CD4, cells/mm^3^, mean (95% CI)	221 (218–224)	209 (206–213)	243 (237–250)	227 (218–235)	235 (223–247)
Baseline CD4, cells/mm^3^, mean (95% CI)^b^	220 (117–223)	210 (206–213)	240(234–247)	225 (216–233)	231 (220–244)
Baseline CD4-T-cell count, cells/mm^3^, n (%)					
< 200	8393 (51.1)	5126 (54.7)	1549 (46.5)	1150 (47.0)	570 (44.0)
200–349	3628 (22.1)	2032 (21.7)	776 (23.3)	528 (21.6)	292 (22.5)
350–499	1550 (9.4)	818 (8.7)	361 (10.8)	228 (9.3)	143 (11.0)
≥ 500	1213 (7.4)	606 (6.5)	316 (9.5)	186 (7.6)	105 (8.1)
Missing	1645 (10.0)	775 (8.3)	327 (9.8)	357 (14.6)	186 (14.4)

*HIV* human immunodeficiency virus, *SD* standard deviation, *IQR* interquartile range

^a^Includes mother to child HIV transmission, intravenous drug use, men who have sex with men and unknown

^b^Missing CD4 T-cell counts computed using multiple imputation methods

### Prevalence of Kaposi Sarcoma

There were 97 documented cases of KS, with an overall crude KS prevalence of 5.9 per 1,000 (95% CI 4.8–7.2 per 1000). Thirty-three cases (34%) of KS had documented histological confirmation. The prevalence of KS was twice as high in males compared to females (9.2 (95% CI 6.7–11.7) per 1000 versus 4.2 (95% CI 2.9–5.4) per 1000).

### Trends in Kaposi Sarcoma

**Fig. 2:**
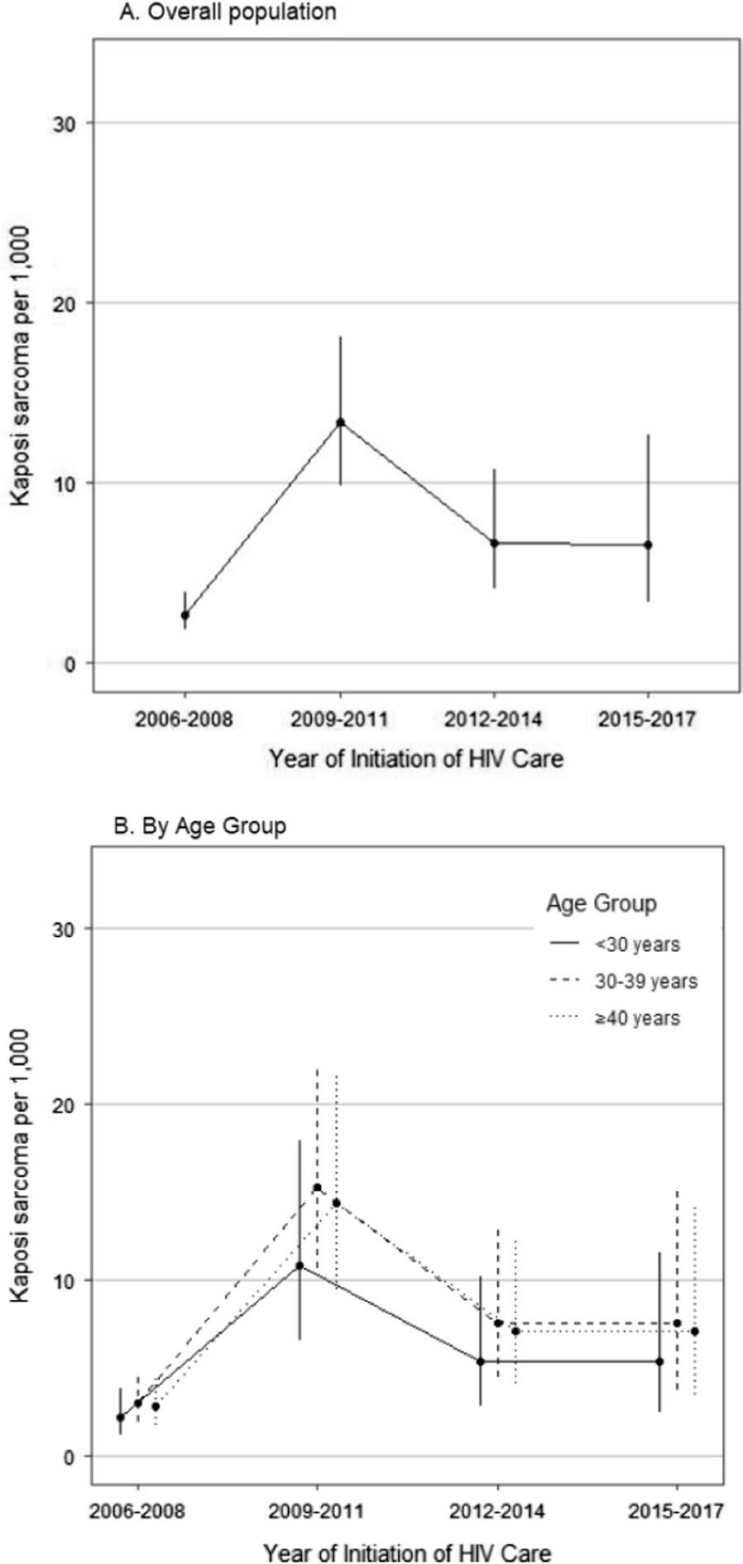

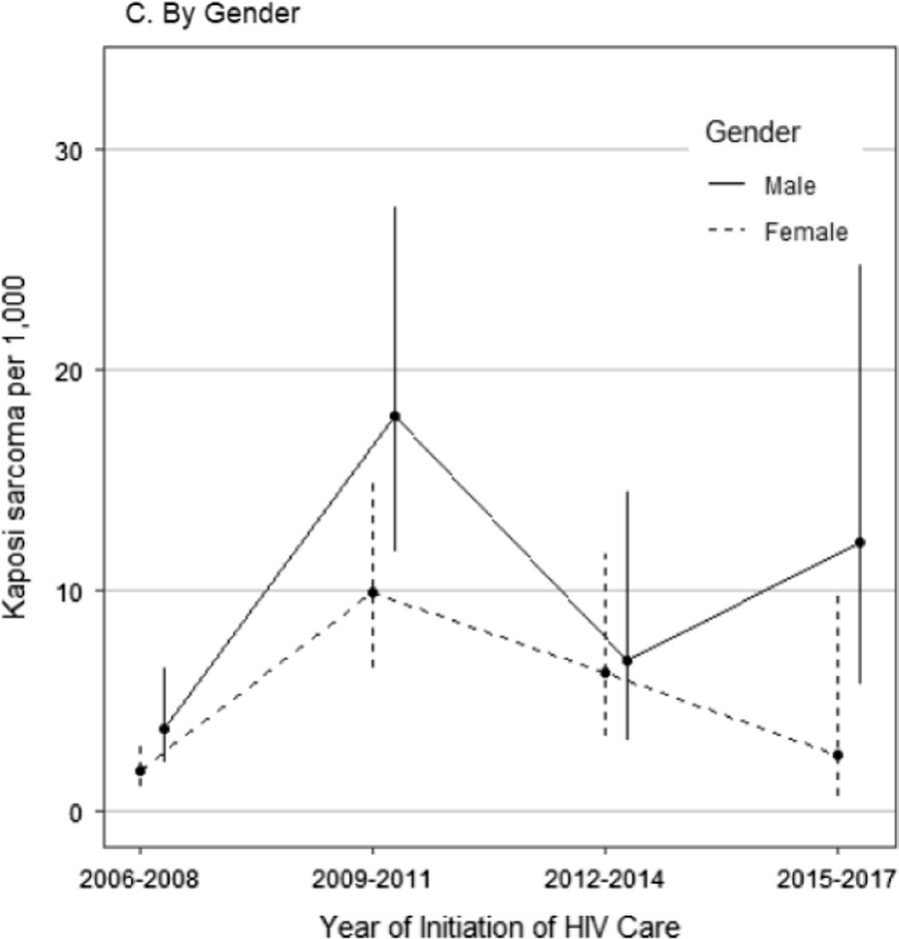
Trends in Kaposi Sarcoma prevalence among adults newly enrolling for HIV care in Jos, Nigeria (2006–2017). [Pairs testing (**A**): 2006–2008 significantly lower than rest; 2009–2011 significantly higher than 2012–2014 but not 2015–2017 (relatively flat trend)]. *HIV* human immunodeficiency virus

The crude prevalence rates per 1,000 persons for 2006–2008, 2009–2011, 2012–2014, and 2015–2017 respectively were: 2.7 (95% CI 1.7–3.9), 13.5 (95% CI 9.5–17.4), 6.9 (95% 3.6–10.2), and 6.9 (95% CI 2.4–11.5). In the analyses adjusted for age and sex, the prevalence of KS was lowest in 2006–2008, with no significant change in subsequent periods (Tables 2, 3). KS trends in the different age groups (< 30, 30–39, and ≥ 40 years) were similar to that of the whole population. Although patients < 30 years had a lower prevalence of KS, the difference was not statistically significant (overlapping confidence intervals) (Fig. 2A, B).

**Table 2:** Factors associated with Kaposi Sarcoma at HIV care enrollment in Jos, Nigeria (2006–2017)

Characteristics	Unadjusted analysis		Adjusted analysis	
	OR (95% CI)	*P* value	OR (95% CI)	*P* value
Year of initiation of HIV care				
2006–2008	Reference		Reference	
2009–2011	4.92 (3.03–7.98)	< 0.001	5.07 (3.12–8.24)	< 0.001
2012–2014	2.51 (1.36–4.63)	0.003	2.50 (1.35–4.62)	0.003
2015–2017	2.51 (1.17–5.36)	0.018	2.47 (1.15–5.30)	0.020
Sex				
Male	Reference		Reference	
Female	0.45 (0.30–0.67)	< 0.001	0.53 (0.34–0.82)	0.004
Age group, years				
< 30	Reference		Reference	
30–39	1.76 (1.03–3.03)	0.038	1.43 (0.82–2.48)	0.209
≥ 40	1.87 (1.07–3.29)	0.028	1.34 (0.73–2.43)	0.342
Baseline CD4 T-cell count, per 100 cells/mm^3^ increase	0.85 (0.74–0.97)	0.02	0.87 (0.76–0.99)	0.04

*HIV* human immunodeficiency virus, *OR* odds ratio

**Table 3:** Pairwise comparison of the prevalence of Kaposi Sarcoma among adults newly enrolling for HIV care in Jos, Nigeria: 2006–2017

Year of initiation of HIV care	OR	SE	z-ratio	*P* value	Tukey adjusted *p* value
(2006–2008)/(2009–2011)	0.195	0.0485	− 6.579	< .0001	< 0.0001
(2006–2008)/(2012–2014)	0.397	0.1243	− 2.951	0.0032	0.0167
(2006–2008)/(2015–2017)	0.4	0.1554	− 2.358	0.0184	0.0854
(2009–2011)/(2012–2014)	2.031	0.5827	2.469	0.0136	0.0649
(2009–2011)/(2015–2017)	2.046	0.7523	1.948	0.0514	0.2081
(2012–2014)/(2015–2017)	1.008	0.4176	0.019	0.9852	1.0000

*HIV* human immunodeficiency virus, *OR* odds ratio, *SE* standard error (analysis adjusted for age and sex)

Overall, KS prevalence was lower in women compared to men (Table 2, adjusted OR 0.52, *p* < 0.01), but this difference was not statistically significant in the trend analysis. However, the trajectory of KS in men appeared to be different from that of women (Fig. 2C). Following a peak in 2009–2011, KS prevalence steadily declined in women in subsequent periods (2012–2014, 2015–1017). While KS trajectory for men was similar to that of women through 2012–2014, KS prevalence in men trended higher in the most recent period (Fig. 2C). Independent of CD4 T-cell count, the prevalence of KS was higher in males compared to females, but this difference was only statistically significant at CD4 T-cell counts between 200 and 500 cells/mm^3^.

### Sensitivity analyses

Trends in annual prevalence of KS using restricted cubic splines analysis showed an increase in KS prevalence from 2006 through 2009, but a gradual, yet insignificant decline from 2008 through 2017 (Additional file 1: Figure S1). Similarly, join point regression suggests similar results, where prevalence rose from 2006 to 2009, and remained relatively flat for the rest of the observation period [join point 2009.9; slope before join point 0.66 (95% CI 0.33–0.88); slope after joint point − 0.12 (95% CI − 0.26 to 0.10)].

Analysis of CD4 T-cell count at enrollment using restricted splines shows an increase in CD4 T-cell count from 2006 through 2010, with CD4 T-cell counts declining in more recent years (Additional file 1: Figure S2).

## Discussion

Among 16,431 Nigerian HIV-positive patients new to care, we found an initial increase in KS prevalence from 2006 to 2009, with a plateau from 2010 through 2017. KS prevalence did not decline significantly in the overall population or in any patient sub-population. Although KS prevalence in men increased from 2015 to 2017, the KS trend in men was not statistically different from that observed among women. Overall, male sex and low CD4-T cell count were independently associated with increased odds for KS. The odds for KS was 48% (adjusted OR 0.52, 95% CI 0.34–0.80, *p* < 0.01) lower in females compared to males, and every 100 cell/mm^3^ increase in CD4 T-cell count reduced the odds for KS by 13% (adjusted OR 0.87, 95% CI 0.76–0.99, *p* = 0.04).

Similar to what we found, recent studies from low- and middle-income countries (LMICs) suggest that, despite significant expansion of HIV programs in these regions, KS may remain an important cause of morbidity and mortality among patients newly enrolling for HIV care [27–29]. A recent multinational study across Latin American countries reported that the risk for KS was 41% lower in 2005 compared to 2010 (adjusted HR 0.59, 95% CI 0.40–0.87), and 70% higher in 2012 (adjusted HR 1.70, 95% CI 1.37–2.09) [28]. By 2015, the risk for KS had declined to rates similar to that of 2010 (adjusted HR 1.83, 95% CI 0.98–3.44) [28]. In another study from East Africa, Semeere et al. [6] reported that the prevalence of KS among adults enrolled for HIV care at an HIV clinic in Mbara, Uganda was relatively unchanged from 2007 to 2012. However, in another HIV program in Western Kenya, KS prevalence declined during the same period (2007–2012) [6]. Unlike reports from LMICs, most studies from high-income countries report a significant decline in KS in patients receiving HIV care since the introduction of ART [23, 30, 31]. However, underserved populations in high income continue to have high incidence of KS in the current ART era, suggesting a link between HIV-associated KS and access to heathcare [3, 32].

A major driver of KS risk among patients newly enrolling for HIV care in LMICs in the contemporary ART era is late initiation of HIV care [33–36]. Several factors could contribute to late presentation for HIV care. These include HIV stigma, lack of access to healthcare facilities due to financial or geographical barriers and prevailing HIV treatment guidelines [35, 37]. From 2006 to 2009, the 2005 Nigerian HIV treatment guideline was in effect, and it recommended ART initiation in adults with CD4 T-cell count ≤ 200 cell/mm^3^ or WHO HIV stage 4 disease or WHO HIV stage 3 disease with CD4 T-cell count < 350 cells/mm^3^. In 2010 adults with CD4 T-cell count < 350 cells/mm^3^ irrespective of WHO HIV stage or the presence of WHO HIV stage 3 or 4 HIV disease, became eligible for ART [14]. In 2014, ART eligibility was further expanded to patients with a CD4 T-cell count < 500 cells/mm^3^, HIV neuropathy, or coinfection with hepatitis B virus. By 2016, all patients with HIV, irrespective of CD4 T-cell count, were eligible for ART [38, 39].

Low CD4 T-cell counts, indicative of late initiation of HIV care, significantly increases the risk for HIV-associated KS [4, 40, 41]. Studies have shown that as CD4 T-cell counts fall below 350 cell/mm^2^, the risk HIV-associated KS becomes significantly higher [23, 40]. In our study, the mean CD4 T-cell count throughout the study period was less than 250 cells/mm^3^. The percentage of patients presenting with a CD4 T-cell count below 350 cell/mm^3^, however, declined by 10% between 2006–2008 (76%) and 2015–2017 (66%). While this shows that progress is being made, the majority of patients initiating HIV care in the current ART era present late to care, which puts them at an increased risk for opportunistic diseases, such as KS. While late presentation to HIV care is a global problem, the burden is disproportionately borne by LMICs [33, 36, 37]. While around 30–50% of patients with HIV present late to care in high-income countries, this percentage is as high as 60–80% in LMICs [33, 36, 37, 42].

In addition to low CD4 T-cell count at entry to care, a high prevalence of HHV-8 in Nigeria [43], as well as other countries in SSA [43], puts the populations in these countries at increased risk for KS. Nigeria is classified as a hyperendemic region for HHV-8 [43]. The prevalence of HHV-8 in Nigerians with HIV is between 60 and 80% [44, 45]. HHV-8 is a necessary factor for the development of KS [25]. Currently, the epidemiology of HHV-8 is not fully understood and there are no recommended interventions against HHV-8 [25].

**Fig. 3:**
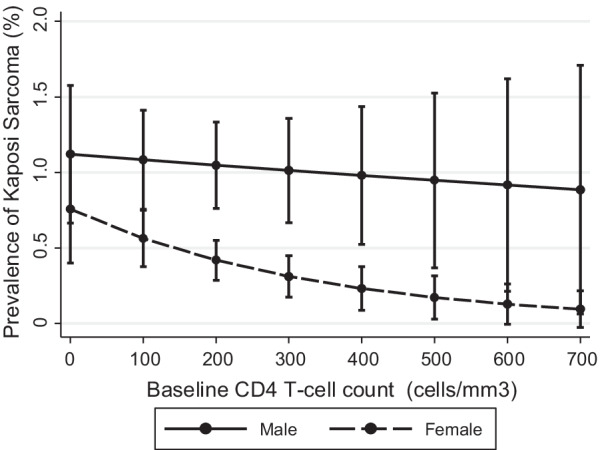
Prevalence of Kaposi Sarcoma at HIV care initiation in adults at the Jos University Teaching Hospital HIV Clinic in Jos, Nigeria (2006–2017), stratified by CD4 T-cell count at HIV care initiation and sex. (Analysis adjusted for age; bars show 95% confidence intervals). *HIV* human immunodeficiency virus

We found a higher risk of KS in men, and this was independent of age or CD4 T-cell count. A higher risk of KS in men has been reported by other studies in patients with HIV-associated KS and those with classical KS (not associated with KS) [1, 4, 46, 47]. The reasons for the higher risk of KS in men is likely multifactorial. In North America and Europe cohorts, the higher prevalence of KS in men has been attributed to the higher risk of co-occurrence of HIV and HHV-8 in men who had sex with men (MSM), who constituted the largest HIV risk group [41, 48]. In Nigeria and most of SSA, heterosexual sexual activity is the major route of HIV transmission, yet many studies have still reported a higher KS prevalence in males, suggesting that there are other unexplained mechanisms [4, 46, 47]. Some studies have attributed the higher risk of KS in men to their older age and more advanced HIV disease at the time of entry to care [50]. Yet, after adjusting for age and CD4 T-cell count, there was still a residual risk for KS in men, suggesting other factors may be at play [50]. In addition to the higher risk of KS in men, we observed a divergence in KS trends between men and women in the most recent observation period. While KS prevalence declined steadily in women since the peak in 2009–2011, it appears there may be a rebound in men, in 2015–2017. While sex differences in KS trends were not a primary objective of our study, one hypothesis proposes that reduced funding to HIV programs in several African countries, including Nigeria [51], may have disproportionately affected interventions targeted at improving HIV diagnosis and linkage to care [52–54]. Since men are underrepresented in HIV prevention, testing, and treatment in SSA, they are more likely to be adversely affected by funding cuts affecting these interventions [52–54]. More studies will be needed to better characterize gender differences in KS trends among patients newly enrolling for HIV care in our setting (Fig. 3).

The findings of this study need to be interpreted in the context of its limitations. First, this is a single-center study from a tertiary health facility, which is a center for excellence for HIV care. The study population may, therefore, differ from those of patients initiating HIV care in other facilities in Nigeria. Also, not all cases of KS were histologically confirmed, which may lead to misclassification and errors in the estimate of KS prevalence. Furthermore, since we analyzed routinely collected clinical data, unmeasured factors, such as the training of health providers, may have influenced recognition and documentation of KS cases. Although rare, patients with visceral KS alone may have been missed due to lower resources for diagnostic testing, such as CT scans, bronchoscopy, and endoscopy, leading to an underestimation of KS prevalence. Also, since the study was limited to adults initiating HIV care, the results may not provide a reliable estimate of changes in the prevalence of HIV-associated KS in the community. Lastly, we did not have data on HHV-8 status, because routine screening for HHV-8 is not recommended for people with HIV. Thus, we were unable to account for the impact of HHV-8 on KS trends in our population.

Despite these limitations, our study highlights the ongoing burden of KS among patients newly enrolling in HIV care in the contemporary ART era in Nigeria. The period covered by our study made it feasible to evaluate changes in KS prevalence during the expansion of Nigeria’s HIV program. Though routinely collected clinical data was utilized, the HIV clinic had rigorous protocols to maintain a high-quality database, which is routinely used for research. In addition, the study population typifies the hospital-based HIV treatment program where the majority of patients with HIV in the country received care [55, 56]. Finally, recent funding support from the US National Institute of Health has also supported the validation of cancer cases within the database, which improved the accuracy of our findings.

In conclusion, despite the significant expansion of Nigeria’s HIV program, KS is yet to decline among patients initiating HIV care. Severe immunosuppression, which persists in this population, most likely contributes to the KS risk. Given the high prevalence of HHV-8 in Nigeria and the absence of effective antiviral agents or vaccines for HHV-8, timely HIV diagnosis and prompt linkage to care remains the most effective approach to reducing KS risk in the pre-ART population. HIV self-testing, which was introduced in Nigeria in 2019 [57], as well as recent interventions focused on community ART, are steps in the right direction [12, 58]. Continued surveillance will be needed to evaluate the impact of these interventions on the KS risk among patients initiating HIV care.

## Supplementary Information

**Additional file 1: Figure 1**. Kaposi Sarcoma prevalence among adults who initiated HIV care from 2006-2017 in Jos, Nigeria. (Model adjusted for year of enrollment, age, and sex. Year and age were expressed using restricted cubic splines with knots at years 2007, 2008 and 2011, and at ages 28, 34, and 40). HIV: Human Immunodeficiency Virus; **Figure 2**. Trends in CD4 T-cell count among adults who initiated HIV care from 2006-2017 in Jos, Nigeria. (Model adjusted for year of enrollment, age, and sex. Year and age were expressed using restricted cubic splines with knots at years 2007, 2008 and 2011, and at ages 28, 34, and 40). HIV: Human Immunodeficiency Virus.

## Data Availability

All study data will be made available by the first author upon request.

## Abbreviations

AIDS: Acquired Immune Deficiency Syndrome
ART: Antiretroviral therapy
HIV: Human Immunodeficiency Virus
IQR: Interquartile range
SD: Standard deviation
SSA: Sub-Saharan Africa
WHO: World Health Organization
