# Supplementary material for: Analyses of Kaposi Sarcoma trends among adults establishing initial outpatient HIV care in Nigeria: 2006–2017
Source: Infect Agent Cancer. 2022 Mar 21;17:10. doi: 10.1186/s13027-022-00424-4 (PMC8935748; doi:10.1186/s13027-022-00424-4)
Supplement: Supplementary file 1 — Additional file 1: Figure 1. Kaposi Sarcoma prevalence among adults who initiated HIV care from 2006-2017 in Jos, Nigeria. (Model adjusted for year of enrollment, age, and sex. Year and age were expressed using restricted cubic splines with knots at years 2007, 2008 and 2011, and at ages 28, 34, and 40). HIV: Human Immunodeficiency Virus; Figure 2. Trends in CD4 T-cell count among adults who initiated HIV care from 2006-2017 in Jos, Nigeria. (Model adjusted for year of enrollment, age, and sex. Year and age were expressed using restricted cubic splines with knots at years 2007, 2008 and 2011, and at ages 28, 34, and 40). HIV: Human Immunodeficiency Virus. [file 13027_2022_424_MOESM1_ESM.docx]

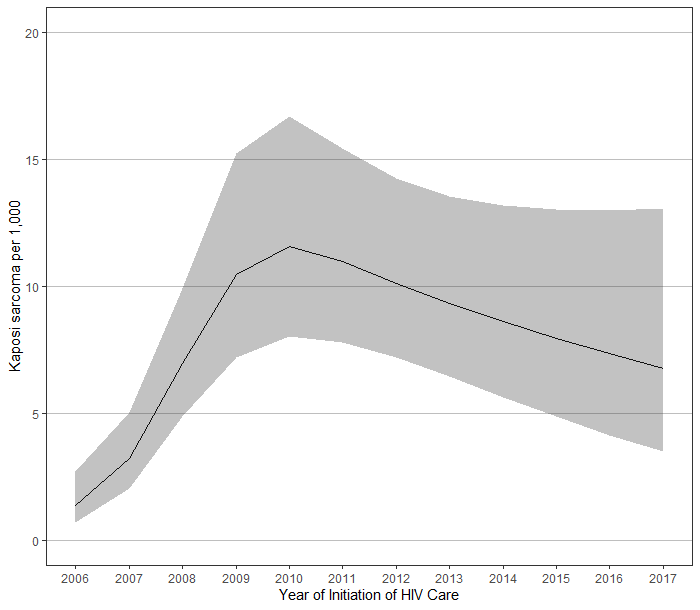


**Supplemental Figure 1**: Kaposi Sarcoma prevalence among adults who initiated HIV care from 2006-2017 in Jos, Nigeria

(Model adjusted for year of enrollment, age, and sex. Year and age were expressed using restricted cubic splines with knots at years 2007, 2008 and 2011, and at ages 28, 34, and 40)

HIV: Human Immunodeficiency Virus


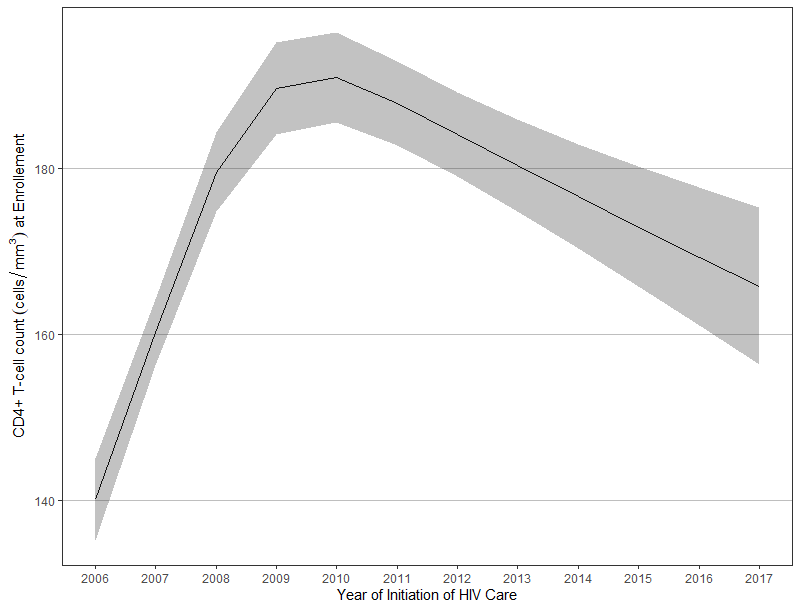


**Supplemental Figure 2**: Trends in CD4 T-cell count among adults who initiated HIV care from 2006-2017 in Jos, Nigeria

(Model adjusted for year of enrollment, age, and sex. Year and age were expressed using restricted cubic splines with knots at years 2007, 2008 and 2011, and at ages 28, 34, and 40)

HIV: Human Immunodeficiency Virus
